# Unveiling macrophage dynamics and efferocytosis-related targets in diabetic kidney disease: insights from single-cell and bulk RNA-sequencing

**DOI:** 10.3389/fimmu.2025.1521554

**Published:** 2025-02-19

**Authors:** Binshan Zhang, Yunqi Wu, Zhongli Wang, Suhua Gao, Hongyan Liu, Yao Lin, Pei Yu

**Affiliations:** ^1^ National Health Commission (NHC) Key Lab of Hormones and Development and Tianjin Key Lab of Metabolic Diseases, Tianjin Medical University Chu Hsien-I Memorial Hospital & Institute of Endocrinology, Tianjin, China; ^2^ Department of Nephrology & Blood Purification Center, The Second Hospital of Tianjin Medical University, Tianjin, China

**Keywords:** single-cell RNA sequencing, diabetic kidney disease, macrophages, efferocytosis, inflammation

## Abstract

**Background:**

Chronic inflammation and immune imbalance mediated by macrophages are considered pivotal in diabetic kidney disease (DKD). The study aims to clarify the macrophage heterogeneity and phenotype dynamics, and pinpoint critical targets within efferocytosis in DKD.

**Methods:**

Utilizing early human DKD sequencing data, we computed the potential communication between leukocytes and renal intrinsic cells. Subsequently, we scrutinized the single-cell RNA sequencing (scRNA-seq) data from CD45-enriched immune cells, concentrating on the macrophage subsets in DKD. Pseudotime trajectory analysis was conducted to explore cell development. Differential expression genes (DEGs) from macrophage subgroups and bulk RNA-sequencing were used to identify shared hub genes. The NephroseqV5 platform was employed to evaluate the clinical significance, and the expression of key molecules was validated in DKD tissues.

**Results:**

Macrophage infiltration rose in DKD, causing inflammation through the release of chemokines. As time progressed, the number of resident macrophages substantially dropped, with diminishing M1-like and increasing M2-like phenotypes relative to early stages. Further analysis pointed to the most enrichment of macrophage function is the phagosome. We overlapped the DEGs with efferocytosis-related genes and identified key genes, including CD36, ITGAM, and CX3CR1, which exhibited significant correlations with macrophages and T cells. The Nephroseq database revealed that they are associated with proteinuria and renal function. Consistent with the validation set, *in vivo* experiments verified elevated expression levels of key molecules.

**Conclusions:**

In essence, our research elucidated the dynamics in macrophage subtype transitions. It emphasized three pivotal genes as critical modulators of macrophage efferocytosis in DKD, indicating their potential as innovative biomarkers and therapeutic targets.

## Introduction

Type 2 diabetes mellitus, commonly known as T2DM, is a chronic metabolic disorder characterized by elevated blood glucose. Epidemiology reveals that approximately 40% of diabetes individuals develop diabetic kidney disease (DKD), a prevalent microvascular complication (1). The progression of DKD is often insidious and asymptomatic in its early stages, but it later manifests a range of pathological changes, such as thickening of the glomerular basement membrane, mesangial expansion, and nodular sclerosis (2). Superior management of DKD is instrumental in easing the heavy financial burden on the global healthcare system (1). The onset of DKD is multifactorial, encompassing metabolic reprogramming, hemodynamic alterations, and oxidative stress. Substantial evidence suggests that chronic inflammation and immune imbalance could be among the key biological processes driving DKD (3). Hyperglycemia induces changes in the renal metabolic microenvironment, and the precise molecular mechanisms of cell-cell interactions remain to be elucidated.

The inflammatory process entails the influx of immune cells, inflammatory cytokines, and chemokines which attract macrophages and lymphocytes to the kidneys. Macrophage infiltration is a critical juncture in the onset and progression of DKD (4). Administering liposomal clodronate to exhaust macrophages could mitigate renal tissue damage (5). Under physiological conditions, there is a delicate equilibrium between classical M1 and alternative M2 macrophages, but in diabetes, this balance can be disrupted, potentially mediating the exacerbation of inflammatory responses and impaired tissue repair (6). Macrophages, serving as specialized phagocytes of the innate immune system (7), recognize and bind to apoptotic cells, initiating cytoskeletal rearrangements that lead to the efficient clearance of dying cells. This process is essential for maintaining the internal tissue environment and is termed efferocytosis.

It seems that macrophages exhibit defective phagocytic capacity when exposed to diabetes. In neonatal diabetes-prone rats, the function of macrophages to clear apoptotic beta-cells is compromised (8), and macrophages from NOD mice phagocytose fewer apoptotic thymocytes (9). Strategies to enhance efferocytosis may improve inflammation resolution and tissue repair (10). For instance, apoptotic vesicles derived from mesenchymal stem cells can induce macrophage reprogramming towards an anti-inflammatory phenotype in an efferocytosis-dependent manner, restoring liver homeostasis to counteract type 2 diabetes (11). Overexpression of the macrophage receptor MerTK to restore efferocytosis can alleviate atherosclerosis in diabetic ApoE^-/-^ mice (12). Nonetheless, the mechanisms and pivotal targets of efferocytosis in DKD are yet to be clarified.

The single-cell RNA sequencing (scRNA-seq) technology enables the high-resolution revelation of cellular gene expression heterogeneity, depicting the process of cell differentiation, thereby advancing our comprehension of cellular subgroups and molecular mechanisms linked to diseases. The single-cell profiling has uncovered renal myeloid heterogeneity during disease progression and regression. Macrophages can assume various phenotypes based on environmental clues, which can be detrimental or facilitate repair by clearing cellular debris and degrading the extracellular matrix (ECM) (13). Parker C et al. present the transcriptomic landscape of early human diabetic kidneys, showing that gene expression variations in distinct cell types are vital for the activation of immune cells, angiogenesis, and ion transport. Compared to the control group, the leukocytes in diabetic kidney samples rose by approximately 7 to 8 times (14). Macrophages are also a principal immune cell cluster in the diabetic glomerulus, with the M1 phenotype particularly prominent during the early phases (15). Moreover, due to insufficient understanding of resident and infiltrating macrophages in DKD, a study focused on isolating renal immune cells to emphasize the dynamic shifts in macrophage phenotype (16). Yet, few studies have delved into the critical targets of efferocytosis in DKD.

High-throughput transcriptomic sequencing data, coupled with advanced bioinformatics tools, illuminate the intricate interplay between immune cells and renal intrinsic cells. Through this approach, significant clusters of immune cells are identified, and the activation characteristics of macrophage subsets are captured, thereby unraveling the diversity within the macrophage population. By integrating bulk RNA-sequencing data with protein interaction networks and machine learning algorithms, critical molecules involved in efferocytosis in DKD are extracted. The correlation of these key targets with immune infiltration is then analyzed, and their diagnostic and prognostic significance for DKD is evaluated. Ultimately, the validity of these findings is confirmed within diabetic kidney tissue.

## Materials and methods

### Data retrieval

The GSE195799 dataset, procured from the Gene Expression Omnibus (GEO) repository (http://www.ncbi.nlm.nih.gov/geo/), was based on the GPL24247 Illumina NovaSeq 6000 platform (16). The scRNA-seq was conducted on CD45+ immune cells isolated from the kidney of Type 1 diabetic OVE26 mice 3 and 7 months of age. The single nucleus RNA sequencing (snRNA-seq) data matrix GSE131882 (14), encompasses samples from three individuals with early human DKD alongside three control subjects.

The tubulointerstitial transcriptomic data series of human DKD, wherein GSE30122, derived from the GPL571 platform, serves as the training cohort, and includes 10 DKD and 24 control individuals. For subsequent verification, GSE104954 is employed as the external validation, consisting of 17 DKD and 21 control participants.

### Single−cell RNA sequencing analysis

We utilized the R package “Seurat” (version 5.1.0) for the processing of scRNA-seq and snRNA-seq data. In advance of conducting downstream analyses, filtered cells meet the following criteria: cells with gene expression exceeding 200 but less than 10,000, and mitochondrial gene expression under 10%. We employed the FindVariableFeatures function to identify the top 2000 high variability genes. The processes of cell clustering were carried out by the FindNeighbors and FindClusters functions. Additionally, the t-Distributed Stochastic Neighbor Embedding (t-SNE) and Uniform Manifold Approximation and Projection (UMAP) for reducing dimensions were implemented by the RunTSNE and RunUMAP functions.

Cell annotation is a vital procedure of the transcriptomic data, we conducted this by consulting CellMarker 2.0 (http://bio-bigdata.hrbmu.edu.cn/CellMarker/) and previous research. Moreover, the FindAllMarkers function was used to screen differentially expressed genes (DEGs) for each cluster. Multiple volcano graphs were generated to illustrate the significantly altered genes for each cell type.

### Cellular communication pathway

Inter-cellular communication is constant to cope with numerous physiological and pathological triggers. We conducted cellular communication analysis with the CellChat R package (version 1.6.1). Using the CellChatDB database, we determined the likelihood of secreted signaling, ECM-receptor, and cell-cell contact interactions among cell types and highlighted overexpressed ligands and receptors.

### Pseudotime trajectory analysis

With monocle2 (version 2.30.1), we engaged in pseudotime trajectory analysis of scRNA-seq data to delve into the dynamics of gene expression patterns (17). The process involves data preprocessing, selecting key genes, dimension reduction, and the linchpin step is to apply the orderCells function to rebuild the temporal trajectory of cell differentiation or development.

### MacSpectrum profiling of macrophage polarization

Upon interacting with the tissue microenvironment, macrophages do not completely polarize to a solitary phenotype but exist in a dynamic state within various subpopulations (18). MacSpectrum (https://macspectrum.uconn.edu), leveraging single-cell transcriptomics, has devised two algorithms that can discern the heterogeneity of macrophages (19). The Macrophage Polarization Index (MPI) is based on “polarization signature genes” that signify the inflammatory condition, and the Activation-Induced Macrophage Differentiation Index (AMDI) sorts “activation-induced differentiation signature genes” to index the relative stage of differentiation maturity. This stratifies macrophages into M1-like, M2-like, transitional, and pre-activation phenotypes, effectively mapping the changing subpopulation composition.

### Bulk sequencing data preprocessing and DEGs analysis

The sva package was applied to mitigate batch effects, discarding probes without gene symbols and averaging those with multiple symbols. By employing the limma package (20) to identify DEGs (|logFC| > 1 and adjusted p < 0.05), we harnessed ggplot2 and pheatmap to underscore the prominently altered genes about tubulointerstitial injury.

### Functional enrichment analysis

The clusterProfiler package is an indispensable tool for functional enrichment analysis. Gene Ontology (GO) (21) identifies significantly enriched biological processes (BP), cellular components (CC), and molecular functions (MF); and Gene Set Enrichment Analysis (GSEA) (22) calculates the enrichment scores for gene sets, thereby enhancing our comprehension of molecular interactions.

### Screening and validation of hub genes

By mapping DEGs to the STRING database (https://cn.string-db.org/), we integrated established and predicted interactions to build the protein-protein interaction (PPI) network. Cytoscape (version 3.8.0) software enabled the visualization of the PPI network and leveraged the plugin to calculate degree. Furthermore, we selected Least

### Absolute shrinkage and selection operator

(LASSO) regression analysis to select hub genes. Their diagnostic effectiveness was evaluated using receiver operating characteristic (ROC) curve.

The expression of key genes was verified against the external validation dataset GSE104954. Furthermore, we implemented the Nephroseq V5 tool (https://nephroseq.org/) evaluate the correlation between key genes and clinical parameters in DKD patients.

### Establish the diabetic kidney disease mouse model

Male C57BL/6 mice, aged between 8 and 9 weeks, were sourced from Huafukang Animal Center (Beijing, China) and housed in a specific pathogen-free environment with a 12-hour light/dark cycle. Blood glucose and body weight were monitored bi-weekly. The experimental group received an 8-week high-fat diet to induce insulin resistance, followed by intraperitoneal injections of 60mg/kg streptozotocin for five consecutive days to damage pancreatic beta cells. A sustained blood glucose level exceeding 16.7 mmol/L from tail vein monitoring is the criterion for a successful diabetes model. After an additional 12 weeks of high-fat diet feeding, the mice were euthanized, and blood, urine, and kidney tissue samples were collected. The study was approved by the Ethics Committee of Tianjin Medical University Chu Hsien-I Memorial Hospital (Tianjin, China).

### Western blot assay

Upon the addition of RIPA lysis buffer, protease inhibitors, and phosphatase inhibitors to the kidney tissue, the mixture was homogenized and then centrifuged at 12,000 rpm for 10 minutes at 4°C. The resulting supernatant was collected and set aside for subsequent procedures. Protein quantification was performed using a Bicinchoninic Acid protein assay kit. Following this, proteins were separated by molecular weights using sodium dodecyl sulfate polyacrylamide gel electrophoresis. After transferring the isolated proteins onto the PVDF membrane, it was blocked with 5% skim milk at room temperature for 2 hours to block non-specific binding sites. Afterwards, the primary antibody incubation was performed at 4°C overnight, encompassing antibodies for CD36 (A19016, ABclonal, China), ITGAM (ab133357, Abcam, Cambridge, UK), and CX3CR1 (13885-1-AP, Proteintech, China). Following a wash step, the secondary antibody was incubated for 1 hour. After thoroughly treating the membrane with ECL reagent, it was photographed in a gel imaging system, and the bands were analyzed using Image J software.

### Extracting RNA and quantitative polymerase-chain reaction

Kidney tissue RNA was extracted with Trizol reagent, and the purity and concentration were measured. Following reverse transcription into cDNA, the SYBR Green Fast qPCR Mix kit (ABclonal, China) was used to amplify the target genes in a 10μl reaction system. The primer sequences for the target genes are listed in **Supplementary Table S1**. During data processing, GAPDH was utilized as an internal reference, and the 2^-ΔΔCt^ approach was employed for semi-quantitative analysis to compute the relative mRNA expression of the target genes. Each experiment was repeated independently three times.

### Macrophage efferocytosis assay *in vitro*

To induce apoptosis in human proximal tubular epithelial cells, trypsinized cells were irradiated with UV light for 30 minutes. Apoptotic cells were collected by centrifugation, washed with serum-free DMEM, and thoroughly resuspended in Dio fluorescent dye (UElandy). Following a 20-minute incubation, the cells were centrifuged and washed again to yield labeled apoptotic cells. The Raw264.7 macrophages were cultured in low glucose (5.5mM) and high glucose (30mM) for 24 hours. Afterward, they were dyed with Did dye (UElandy). The labeled apoptotic cells and macrophages were co-incubated at a 5:1 ratio for 2 hours, followed by imaging with a fluorescence microscope to assess the engulfment of apoptotic cells. The efferocytosis index, defined as the ratio of macrophages that have engulfed apoptotic cells to the total macrophages count, was used for analysis.

### Statistical analyses

R software (version 4.3.2) and GraphPad Prism (version 10.2.0) were utilized for statistical analyses. Comparison between two groups was conducted using the Wilcoxon or Student’s t-test. A p-value less than 0.05 was considered statistically significant.

## Results

### The profile of human DKD derived from the SnRNA-seq

**Figure 1:**
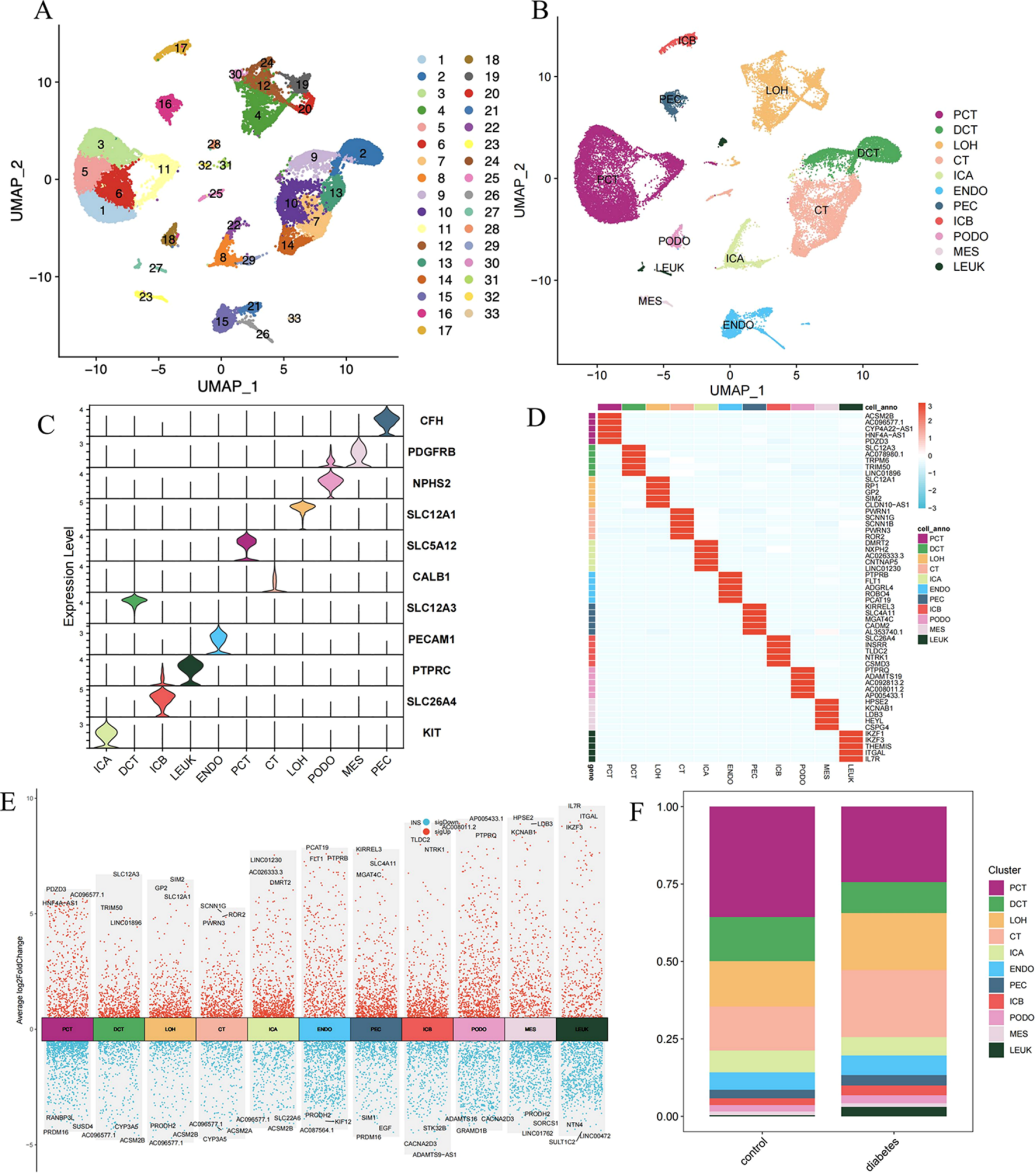
Establishing the cell type in human diabetic kidney disease via snRNA-seq. **(A)** UMAP reduces the dimensionality of data, organizing it into 33 clusters. **(B)** The annotation of all cells yielding 11 cell types, comprising PCT, DCT, LOH, CT, ICA, ENDO, PEC, ICB, PODO, MES, LEUK. **(C)** The violin plot using width to show the average expression level of genes and shape to illustrate the expression variability, thereby visualizing the distribution patterns of marker genes. **(D)** Each cell cluster’s genes are shown in the heatmap, where the shading of colors is based on the logFC values. **(E)** The volcano plots depicting the alterations in gene expression across diverse cell types. **(F)** The distribution of each cell type in the control and diabetes group. UMAP, Uniform Manifold Approximation and Projection; PCT, proximal convoluted tubule; DCT, distal convoluted tubule; LOH, loop of Henle, CT, connecting tubule; ICA, type A intercalated cells; ENDO, endothelial cells; PEC, parietal epithelial cells; ICB, type B intercalated cells; PODO, podocytes; MES, mesangial cells; LEUK, leukocytes.

The GSE131882 dataset comprises kidney samples from three individuals with early human DKD and three control subjects. After discarding low-quality cells, we obtained 12,862 cells from the diabetes group and 17,410 cells from the control group (**Supplementary Figure S1**, **Supplementary Table S2**). Subsequent dimensionality reduction and clustering yielded 33 clusters (**Figure 1A**). Cell annotation, guided by established literature and marker genes, culminating in the identification of 11 cell types: proximal convoluted tubule (PCT), parietal epithelial cells (PEC), loop of Henle (LOH), distal convoluted tubule (DCT), connecting tubule (CT), type A intercalated cells (ICA), type B intercalated cells (ICB), podocytes (PODO), endothelial cells (ENDO), mesangial cells (MES), leukocytes (LEUK) (14) (**Figure 1B**). **Figures 1C, D** present the distinctive features of cell marker genes using violin charts and heat maps. **Figure 1E** displays a volcano plot, which highlights the dynamics of gene expression profiles across various cell types. An upsurge is observed in the expression of IL7R, ITGAL, and IKZF3, simultaneously with a pronounced decrease in the expression of NTN4, SULT1C2, and LINC00472 within leukocytes. Moreover, we noted a reduction in the quantity of certain renal tubular cells and a contrasting rise in the infiltration of leukocytes as DKD progresses (**Figure 1F**).

### Immune cells as the principal catalysts of inflammation and fibrosis in DKD

**Figure 2:**
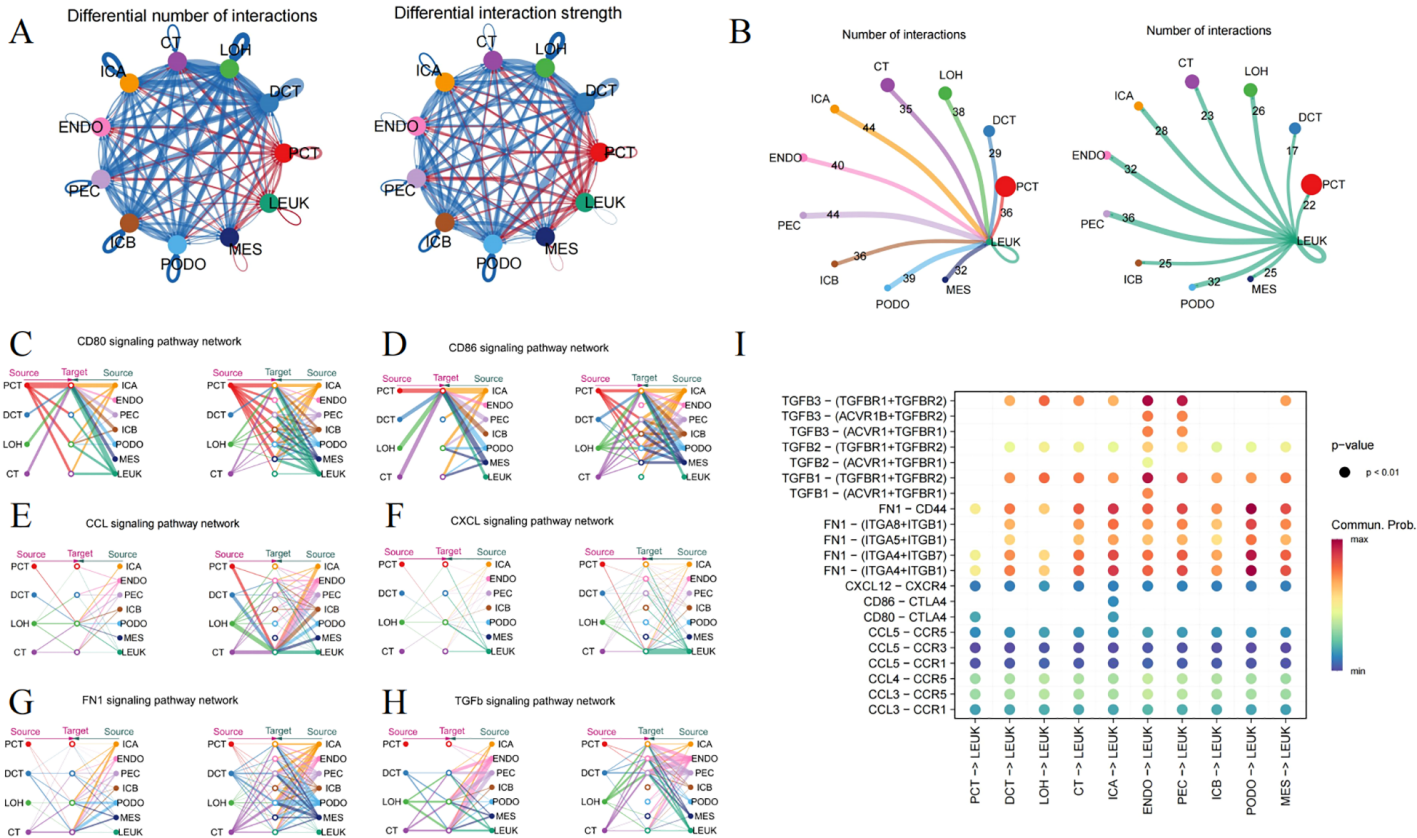
Ligand-receptor interactions surrounding immune cells. **(A)** Circle plot depicting the number and strength of interactions among different cell types, where red indicating an enhancement, and blue denotes a downturn in the DN group relative to the control. **(B)** The frequency of interaction leukocytes interaction with other cells, separately as a ligand and receptor. **(C)** CD80, **(D)** CD86, **(E)** CCL, **(F)** CXCL, **(G)** FN1 and **(H)** TGFb signaling pathway representing through the Hierarchy plot. **(I)** The Dotplot displaying the predicted likelihood of signaling pathways.

By synthesizing secreted signaling, ECM-Receptor, and cell-cell contact, and incorporating prior insights into the interactions, Cellchat forecasts the signaling pathways that mediate cellular communication, enhancing our grasp on how cells behave within tissue environment. As depicted in **Figure 2A**, the strength and count of interactions between leukocytes and glomerular and tubular cells are augmented in DKD versus the control. Immune cell infiltration is essential in mediating diabetic kidney injury (23), as macrophages, operating as both ligands and receptors, communicate with glomerular and tubular cells, leading to glomerulosclerosis and interstitial fibrosis (**Figure 2B**).

CD80 and CD86, serving as markers on macrophages, engage with CD28 or CTLA-4 to govern the immune response through direct cell-cell contact (**Figures 2C, D**). Hyperglycemia leads to an enhanced expression of chemokines in kidney tissue, such as CCL2, CCL3, CCL4, CCL5, and CXCL12, involving in immune cell recruitment and modulating the inflammation (**Figures 2E, F**). Additionally, the FN1 signaling pathway features robust interactions between renal tubules and glomeruli (**Figure 2G**). The TGFΒ pathway also extends its influence on leukocytes (**Figure 2H**). The malfunctioning of the collagen and TGFΒ pathways might contribute to excessive ECM accumulation. **Figure 2I** visualizes the possible interactions of signaling pathways with leukocytes acting as the ligand.

### ScRNA-seq analysis unveils the intricacy of immune cells in the mouse kidney

With significant plasticity, macrophage phenotypes can alter in response to hyperglycemia. Strategically controlling the polarization of macrophages to strike a balance between M1 and M2 could be pivotal for combating DKD. Therefore, we employed scRNA-seq of the diabetic kidneys from OVE26 and WT mice to delve into the distinctive functions and mechanisms of macrophages in the progression of DKD.

**Figure 3:**
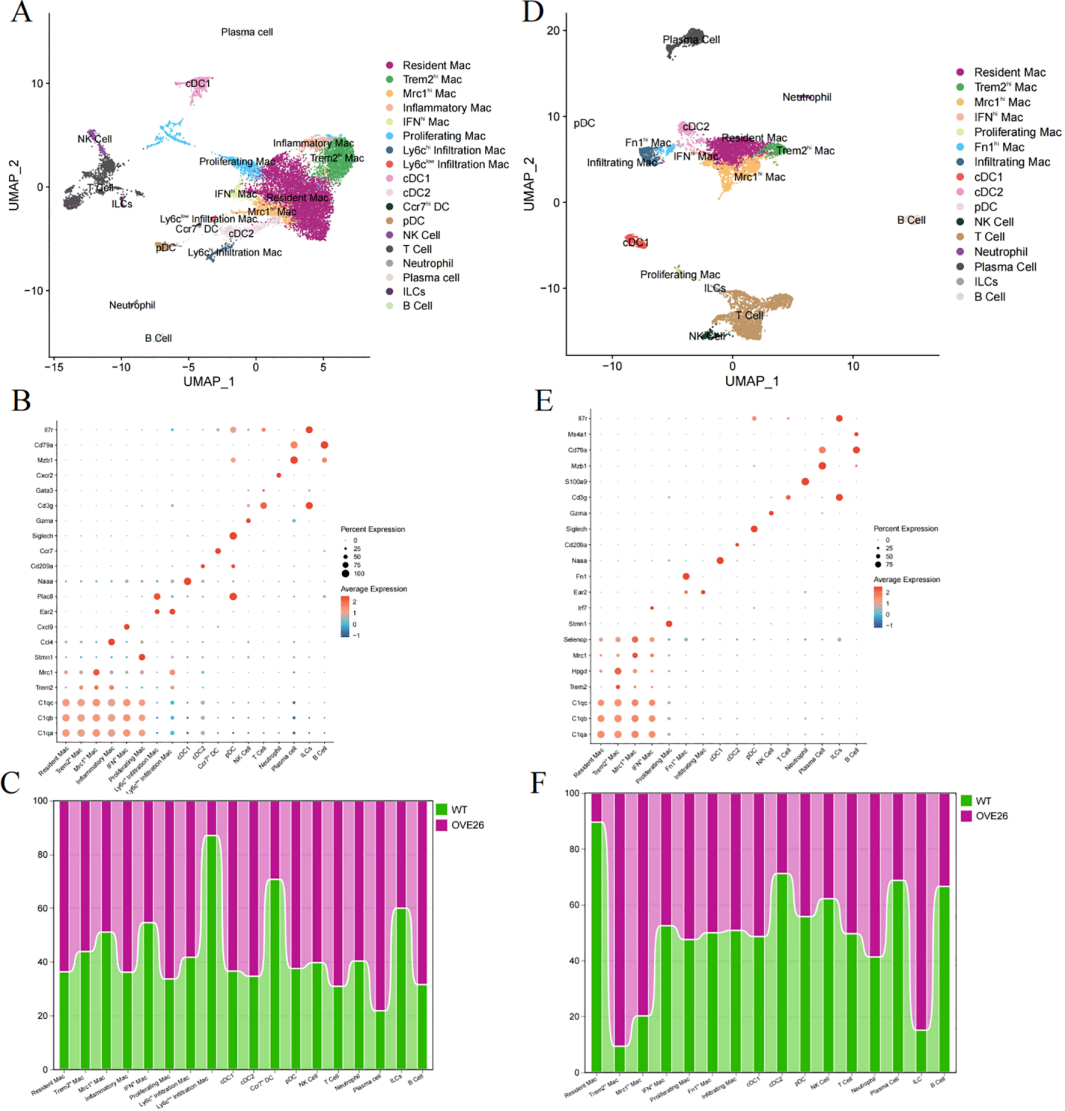
Analysis of immune cell subpopulations in diabetic kidney using single-cell RNA sequencing. **(A–C)** respectively display the UMAP plots **(A)** and dotplots of marker genes **(B)** for CD45+ cells in 3-month-old WT and OVE26 mice, as well as the trend of changes in the proportions of cells between the two groups **(C)**. **(D–F)** sequentially present the UMAP **(D)**, dotplot **(E)**, and proportional change histograms **(F)** of 7-month-old mice. UMAP, Uniform Manifold Approximation and Projection. Mac, macrophage; Trem2^hi^, triggering receptor expressed on myeloid cells 2 high expressing; Mrc1^hi^, Mannose receptor C-type 1 high expressing; IFN^hi^, Interferon gene signature high expressing. DC, dendritic cell; cDC, conventional DC; pDC, plasmacytoid DC; NK cell, natural killer cell; ILCs, innate lymphoid cells.

We selected the GSE195799 dataset to delve into the CD45-positive immune cell subpopulations. CD45+ myeloid cells were isolated from the kidneys of 3-month-old mice. After quality control, data normalization, and dimensionality reduction, the data was categorized into 25 clusters (**Supplementary Figure S2**). By leveraging and integrating characteristics from multiple experimental platforms for the inference of cell functions (13, 16, 24, 25), 18 unique types of immune cells infiltrating the kidney were identified (**Figure 3A**). The most prominent macrophages can be separated into “Resident Mac”, “triggering receptor expressed on myeloid cells 2 (Trem2)-high expressing (Trem2^hi^) Mac”, “Mannose receptor C-type 1 (Mrc-1)-high expressing (Mrc1^hi^) Mac”, “Inflammatory Mac”, “Interferon (IFN) gene signature-high expressing (IFN^hi^) Mac”, “Proliferating Mac”, and “Infiltration Mac”.

First, we referred to these cell surface markers (C1qa, C1qb, C1qc, Lst1, and Cx3cr1) to pick out resident macrophages (25). Building on the shared genes of resident macrophages, five other macrophage subpopulations exhibit different biological roles due to the expression of other outstanding cell markers. Trem2^hi^ and Mrc1^hi^ Mac perform functions like M2 macrophages, including anti-inflammatory, phagocytic, tissue repair, and immunoregulatory functions. Conversely, IFN^hi^ and inflammatory Mac respond to interferon and secrete more pro-inflammatory factors (such as TNF-Α, IL-1Β, IL-6, etc.) and chemokines (such as Ccl3, Cxcl2, Cxcl9, etc.). Proliferating Mac activates genes related to DNA replication, repair, and cell cycle. Infiltration Mac were further subdivided into ly6c^hi^ (Ear2, Ace) and ly6c^low^ (Plac8, Ccl24) subpopulations. Additionally, dendritic cells (DC) were subdivided into cDC1, cDC2, pDC (plasmacytoid DC), and Ccr7^hi^ DC. We also manually annotated clusters into “NK Cell”, “T Cell”, “Neutrophil”, “Plasma cell”, “innate lymphoid cells (ILCs)”, and “B Cell” (**Figure 3B**). As illustrated in **Figure 3C**, in the diabetic kidneys of 3-month-old OVE26 mice, the proportion of resident and inflammatory macrophages slightly increased, whereas the counts of Ly6c^low^ infiltration macrophages, Ccr7^hi^DC, and ILCs displayed a downward trend.

As diabetic kidney disease progresses, 7-month-old OVE26 mice show pronounced glomerular enlargement and mesangial expansion, leading to significant proteinuria (16). Simultaneously, the accumulation of immune cells is evident. Therefore, scRNA-seq analysis of the CD45-enriched myeloid cells helps elucidate the dynamic transformation of macrophages. Following the execution of the analysis procedures (**Supplementary Figure S3**), we identified immune cell subpopulations in the kidneys of 7-month-old mice that are generally like those identified previously, but with a completely different overall proportion (**Figures 3D, E**). Fibronectin 1 (Fn1)-high expressing (Fn1^hi^) Mac also possesses the characteristics of infiltration Mac (Ear2). In the OVE26 group, the proportion of resident macrophages decreased to less than 10%, while the number of Trem2^hi^ and Mrc1^hi^ macrophages with M2 characteristics experienced a sharp rise. Furthermore, DCs, plasma cells, and B cells also diminished (**Figure 3F**).

### MacSpectrum decoding the heterogeneity and dynamics of macrophages in DKD

**Figure 4:**
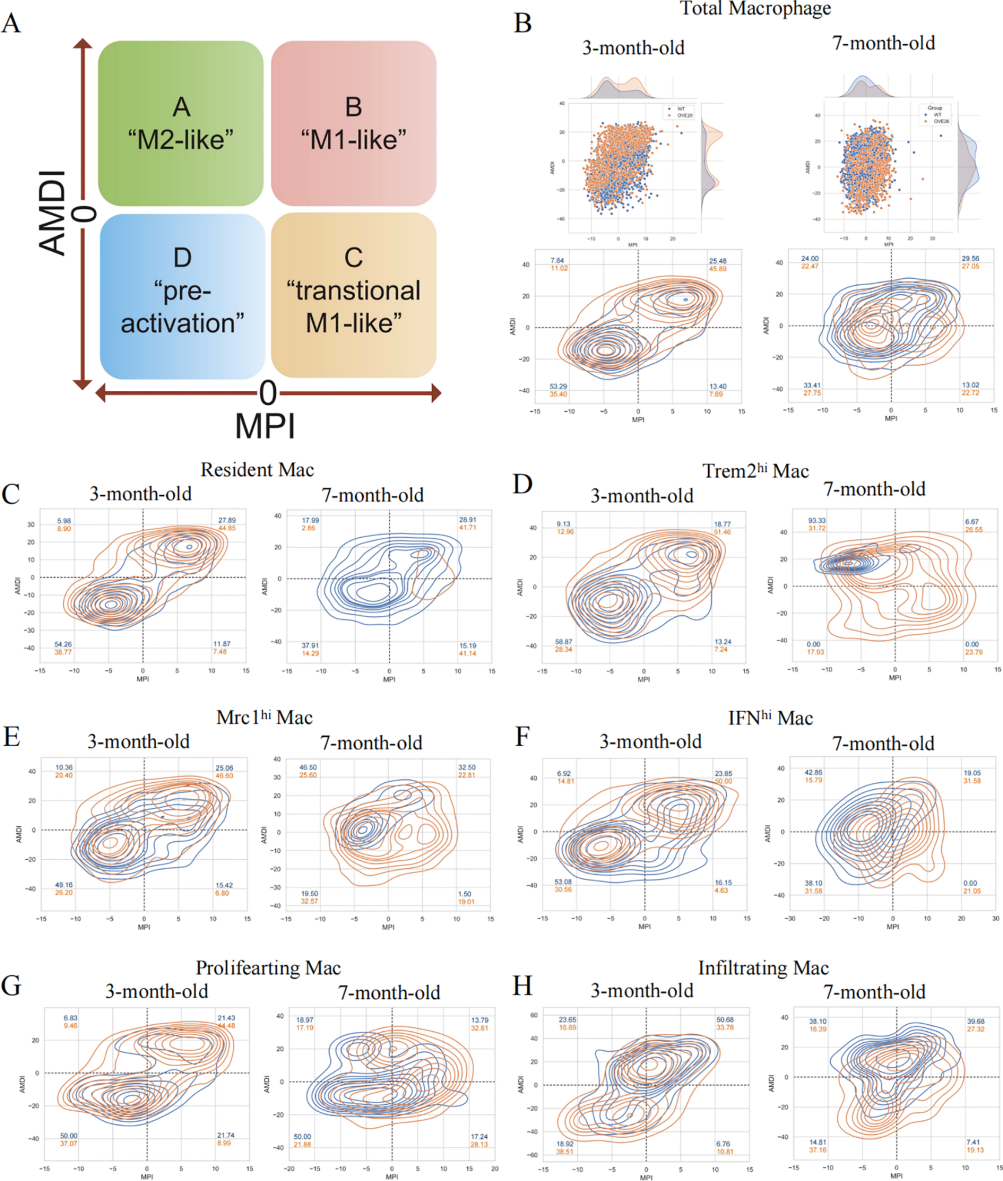
The Macspectrum signatures of renal macrophages in WT and OVE26 mice. **(A)** Macrophages with sustained activation are segmented into four distinct phenotype based on the Macspectrum plot. **(B)** Scatter and contour plots visualizing the distribution patterns of the MPI and AMDI for total macrophages. **(C–H)** Contour plots for multiple macrophage subpopulations showing the proportion of various phenotypes. MPI, macrophage polarization index; AMDI, activation-induced macrophage differentiation index; Mac, macrophage; Trem2^hi^, triggering receptor expressed on myeloid cells 2 high expressing; Mrc1^hi^, Mannose receptor C-type 1 high expressing; IFN^hi^, Interferon gene signature high expressing.

The plasticity of macrophages enables them to respond swiftly, intricately, and diversely under pathological conditions (26). However, the traditional classification does not adequately annotate the intricate features of macrophages. As such, we introduce an innovative and extensive annotation platform, MacSpectrum (19), which is based on two newly devised algorithms: the MPI and AMDI. By precisely mapping each cell to the “inflammatory” or “terminal maturation” status, MacSpectrum achieves a full spectrum and high-resolution annotation of macrophage subpopulations *in vivo*. With these algorithms, MacSpectrum categorizes macrophages into M1-like, M2-like, transitional, and pre-activation phenotypes (**Figure 4A**).

The dynamic changes of activation profiles in the total macrophage population of kidneys of varying ages are displayed in **Figure 4B**. Most macrophages are in the “pre-activation” phenotype across the different stages of the WT group. At 3 months of age, more than half of the macrophages exhibit lower MPI and AMDI. The transformation at 7 months old is reflected in the rise of M2-like macrophages in the WT group (7.84% at 3 months versus 24.00% at 7 months). Additionally, we noted a decline in the M1-like phenotype of macrophages within the OVE26 cohort at later time points (45.89% at 3 months versus 27.05% at 7 months), accompanied by a rise in the M2-like phenotype (11.02% at 3 months versus 22.47% at 7 months) (**Figure 4B**). Upon a meticulous examination of each subset, it was evident that nearly all groups followed a comparable pattern. The results offer additional support for the opinion that macrophages exist in a continuously activated state (**Figures 4C–H**).

**Figure 5:**
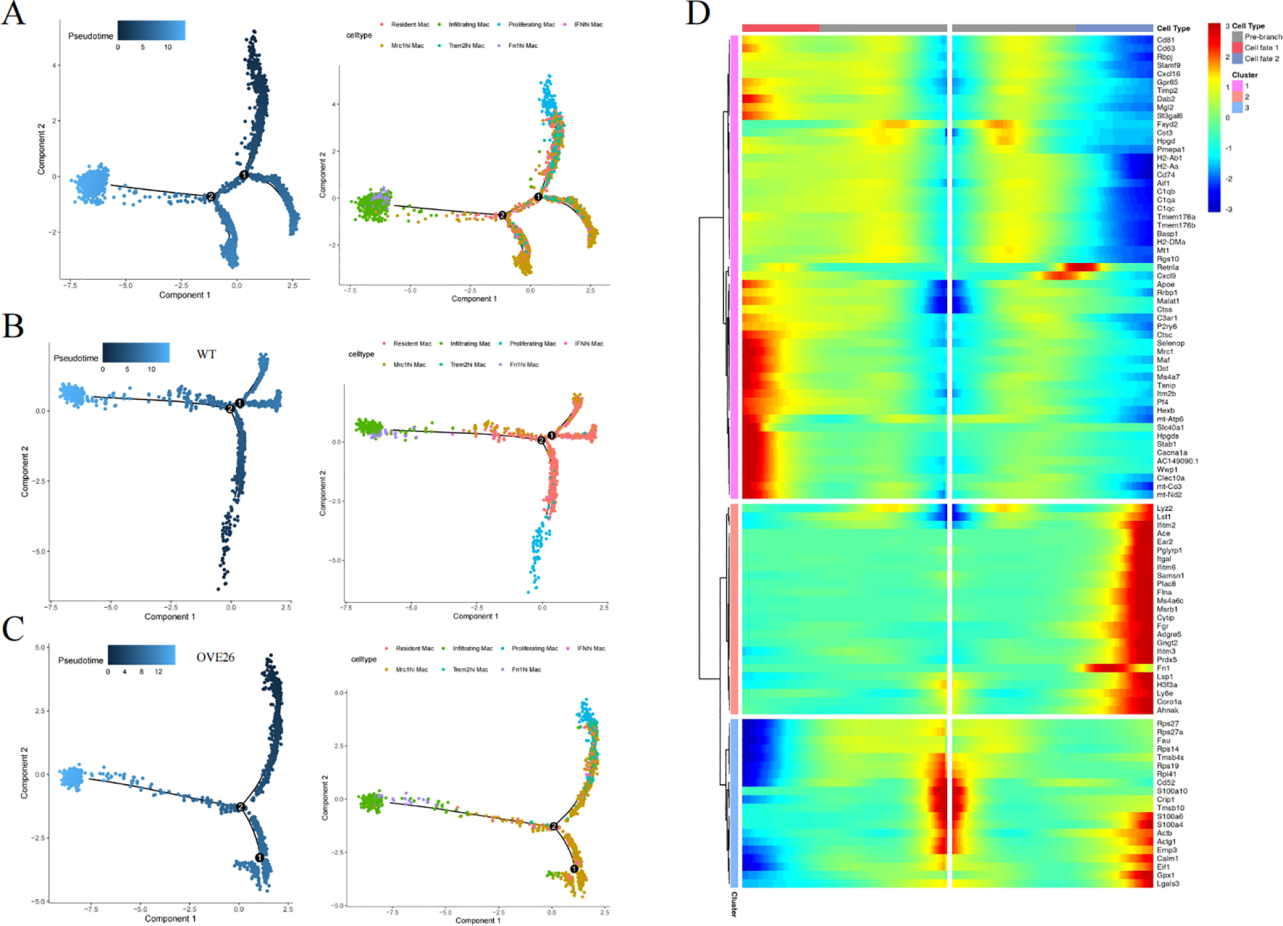
Pseudo-time and trajectory analyses of renal macrophages. **(A)** The trajectory map of macrophage clusters in 7-month-old kidneys features a color scheme based on pseudotime, transitioning from deep to light blue to depict the cellular evolutionary process. The cell differentiation trajectories of the **(B)** WT and **(C)** OVE26 groups. **(D)** The heatmap visualizing how genes that are differentially expressed alter over the trajectory of cell differentiation.

Subsequently, we engaged in pseudo-temporal analysis to reveal the fate trajectories of macrophages within the kidneys of 7-month-old mice, and to reenact the timeline of cellular evolution using monocle2. The trajectory tree orders the cells based on pseudotime, with the deepest color representing the root (**Figures 5A–C**). At branch node 2, the trajectory splits into two cell fates. **Figure 5D** outlines the differential expression of genes across macrophage fates; genes like Apoe, Malat1, Ctsc, and Wwp1 are prominently expressed in fate 1, while Fn1, Ace, Itgal, and Fgr achieve peak levels in fate 2.

### Selecting efferocytosis-related hub genes within macrophages

**Figure 6:**
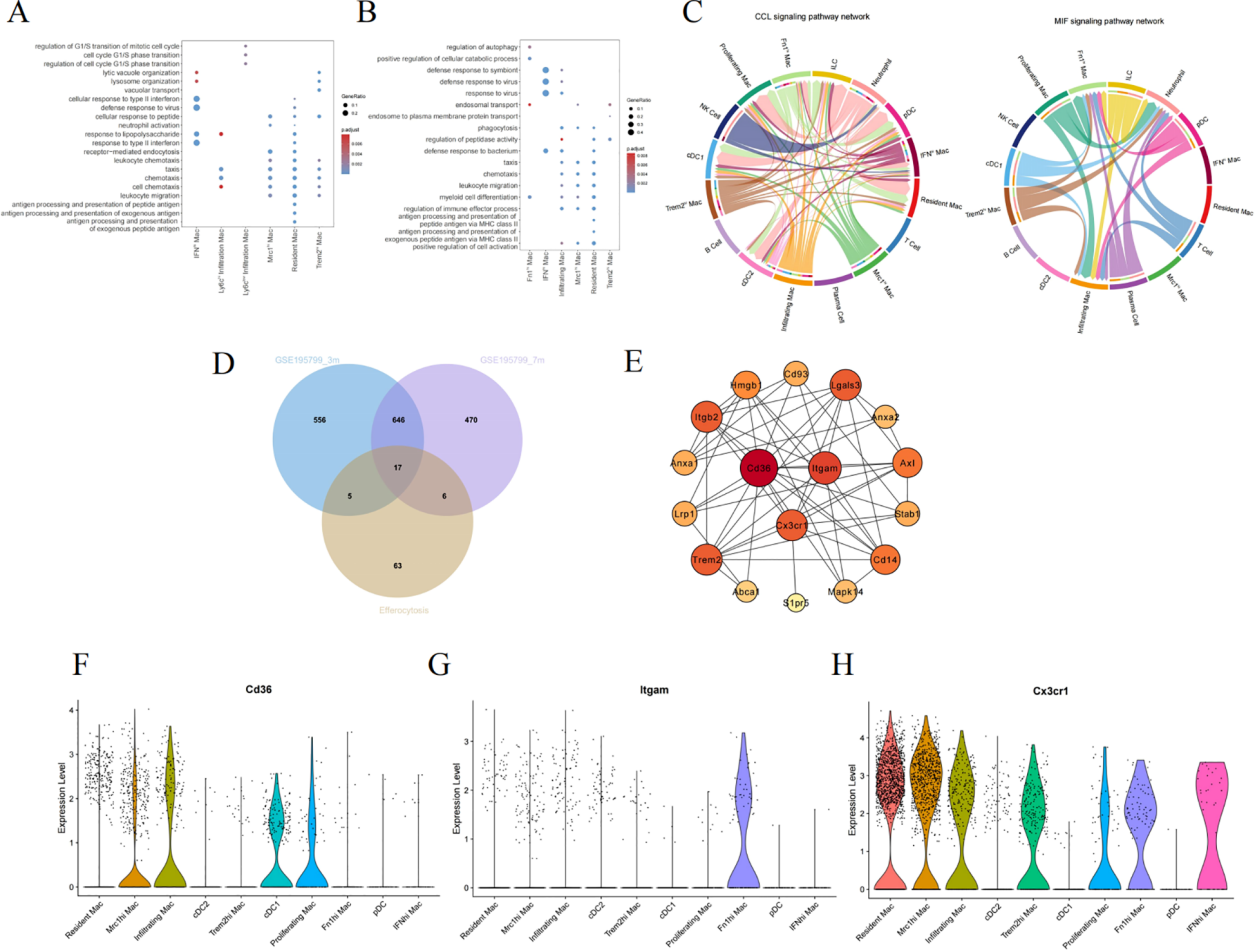
Screening hub genes related to efferocytosis in diabetic kidney macrophages. The GO enrichment analysis of macrophage subpopulations in 3-month-old **(A)** and 7-month-old mice **(B)**. **(C)** The chord diagram about the interaction among renal immune cells. **(D)** The Venn diagram illustrating the intersection between GSE195799 and the gene list of efferocytosis. **(E)** The PPI diagram, where the color varies from light to dark, signifies an ascending order of degree. **(F–H)** Violin diagrams of the distribution of CD36, ITGAM, and CX3CR1 in each cluster. GO, gene ontology; MIF, macrophage migration inhibitory factor; PPI, protein-protein interaction network.

We performed GO enrichment analysis on the DEGs at 3 and 7 months of age to further comprehend the molecular functions of macrophage subpopulations (**Figures 6A, B**). They manifest both general biological functions and pinpoint pathways unique to certain subgroups. As demonstrated by the enrichment analysis results, the BP in the 3-month-old group are primarily centered on regulation of the cell cycle, vacuolar transport, antigen processing and presentation, and receptor-mediated endocytosis (**Figure 6A**). Nevertheless, the 7-month-old mice exhibit significant BP including regulation of autophagy, phagocytosis, leukocyte migration, and chemotaxis. Resident Mac and infiltrating Mac are particularly involved in myeloid cell differentiation and several inflammation-related pathways (**Figure 6A**). Immune cells communicate through direct interactions or secreted mediators, with CCL and macrophage migration inhibitory factor being one of the most prominent signaling pathways (**Figure 6C**).

As inflammation pathways gain prominence in the progression of DKD, M2 macrophages particularly exert anti-inflammatory effects through efferocytosis. Efferocytosis, the process by which macrophages engulf and clear dying cells, prevents the secondary release of harmful substances after cell death, thus facilitating the resolution of inflammation and the repair of tissues (27). To delve deeper into the hubs, We collated a list of genes associated with efferocytosis from literature (**Supplementary Table S3**) (27–29), and subsequently used a Venn diagram to intersect this list with the DEGs of macrophage subpopulations, ultimately yielding 17 genes (**Figure 6D**). Cytoscape was employed to construct a network of protein interactions, where the nodes with greater degrees are colored redder. CD36, ITGAM, and CX3CR1 are among the nodes with higher degrees (**Figure 6E**). **Figures 6F–H** individually depict the expression of the above hub genes within phagocytes, where CD36 is prominently expressed in both Mrc1^hi^ Mac and Infiltrating Mac, while CX3CR1 is expressed across different macrophages.

### Screening and assessing the hubs utilizing bulk RNA-sequencing

**Figure 7:**
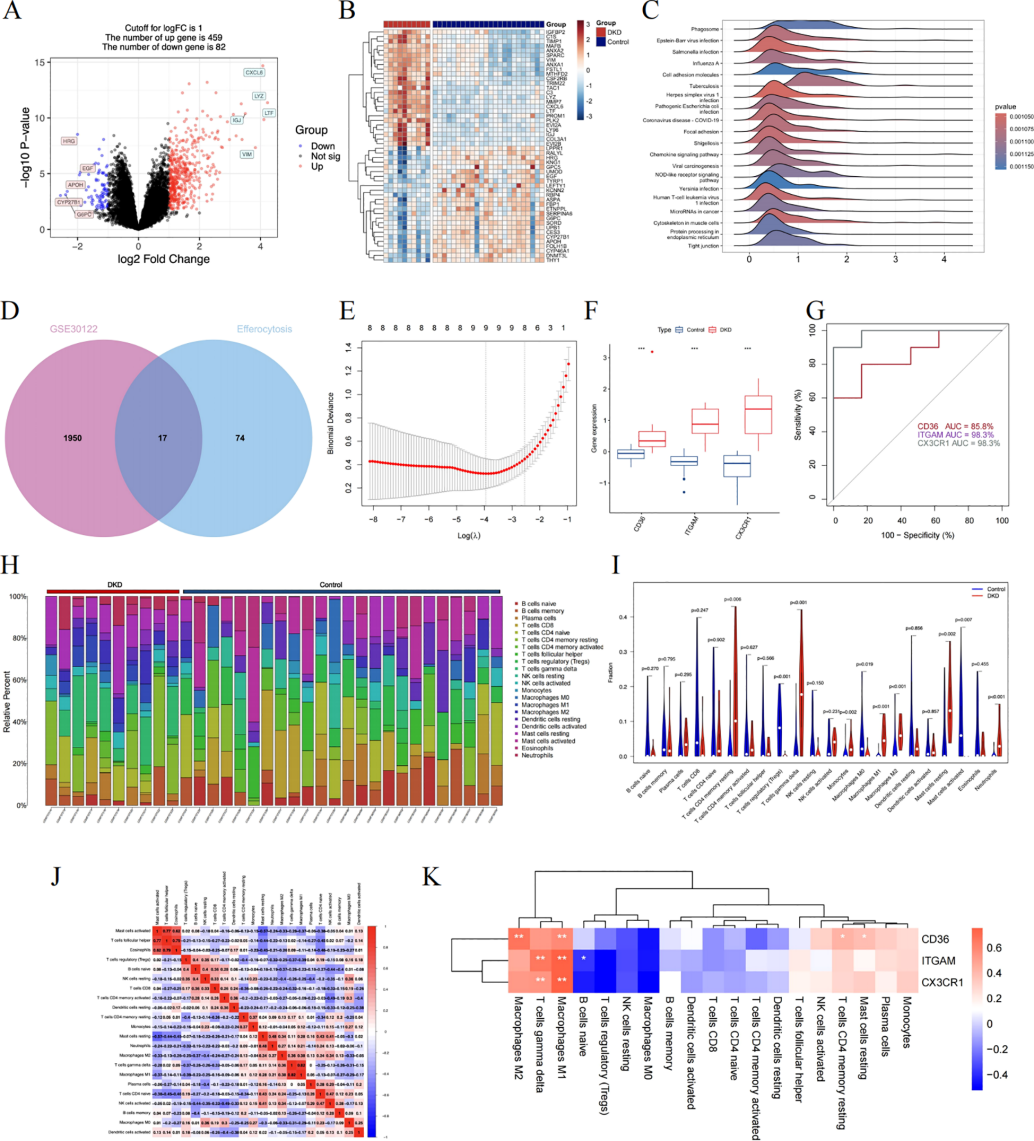
A comprehensive evaluation of the hub genes using bulk RNA sequencing. **(A)** The volcano plot displaying DEG in renal tubulointerstitial tissue. **(B)** The heatmap showing the top 50 genes with notably distinct expression levels between the two groups. **(C)** The GSEA mountain plot showcasing the enriched pathways. **(D)** The shared genes are shown by the Venn diagram. **(E)** The LASSO regression fitting model. **(F)** The boxplot depicting the expression differences of hub genes between the DKD control group. **(G)** The ROC curve in training dataset. **(H)** Stacked histogram identifying each infiltrating immune cell. **(I)** The distribution of 22 different immune cell populations. Data were examined through Wilcoxon tests. **(J)** The interconnectivity of diverse immune cell types. **(K)** The correlation results between hub genes and immune cells. Statistical approaches draw on Spearman’s correlation analysis. DEGs, differentially expressed genes; GSEA, gene set enrichment analysis; LASSO, least absolute shrinkage and selection operator; ROC, receiver operating characteristic. *P < 0.05, **P < 0.01, ***P<0.001.

At the outset, scRNA-seq was used to preliminarily identify candidate genes, which displayed distinctive biological functions in specific cell types. We then focused on the GSE30122 training dataset, leveraging this gene expression profile to uncover the crucial molecules linked to the progression of DKD. We set the logFC cutoff value at 1, yielding 459 upregulated and 82 downregulated DEGs (**Figure 7A**). **Figure 7B** is a heatmap showcasing genes that exhibit significantly varied expression. GSEA analysis highlighted the “phagosome” pathway as the most enriched, alongside other significant pathways such as cell adhesion molecules, chemokine, and NOD-like receptor signaling pathway (**Figure 7C**). We once again intersected the efferocytosis gene list with the DEGs from GSE30122 to identify common genes (**Figure 7D**). Following this, we applied LASSO regression to model fitting, selected, and assessed the diagnostic features for DN (**Figure 7E**). Considering the node count in the PPI network, we ended up with CD36, ITGAM, and CX3CR1 as the final genes of interest. We reviewed the expression levels of the hub genes, which, as shown in **Figure 7F**, exhibited an increasing trend in the DKD group. As shown in **Figure 7G**, the ROC curve illustrates superior performance in diagnosing DKD, especially with ITGAM and CX3CR1 boasting AUC values of 98.3%.

To explore the critical molecule associated with efferocytosis in DKD, the CIBERSORT algorithm is applicable to gauge the abundance of 22 immune cell types in the renal tubulointerstitial tissue (**Figure 7H**). We detected rises in the levels of monocytes, M1 and M2 macrophages, resting mast cells, and neutrophils within the DKD group, while in the control group, there was an upsurge in regulatory T cells, M0 macrophages, and activated mast cells (**Figure 7I**). Additionally, as shown by the correlation heatmap (**Figure 7J**), eosinophils and T cells follicular helper exhibited a significant positive correlation (r = 0.79), and conversely, a negative correlation was observed between M1 macrophages and regulatory T cells (r = -0.39). Among these immune cells, each pivotal gene displayed a positive relationship with M1 macrophages, with CD36 additionally correlating positively with M2 macrophages (**Figure 7K**).

### Validate hub genes and recognize their prognostic significance

**Figure 8:**
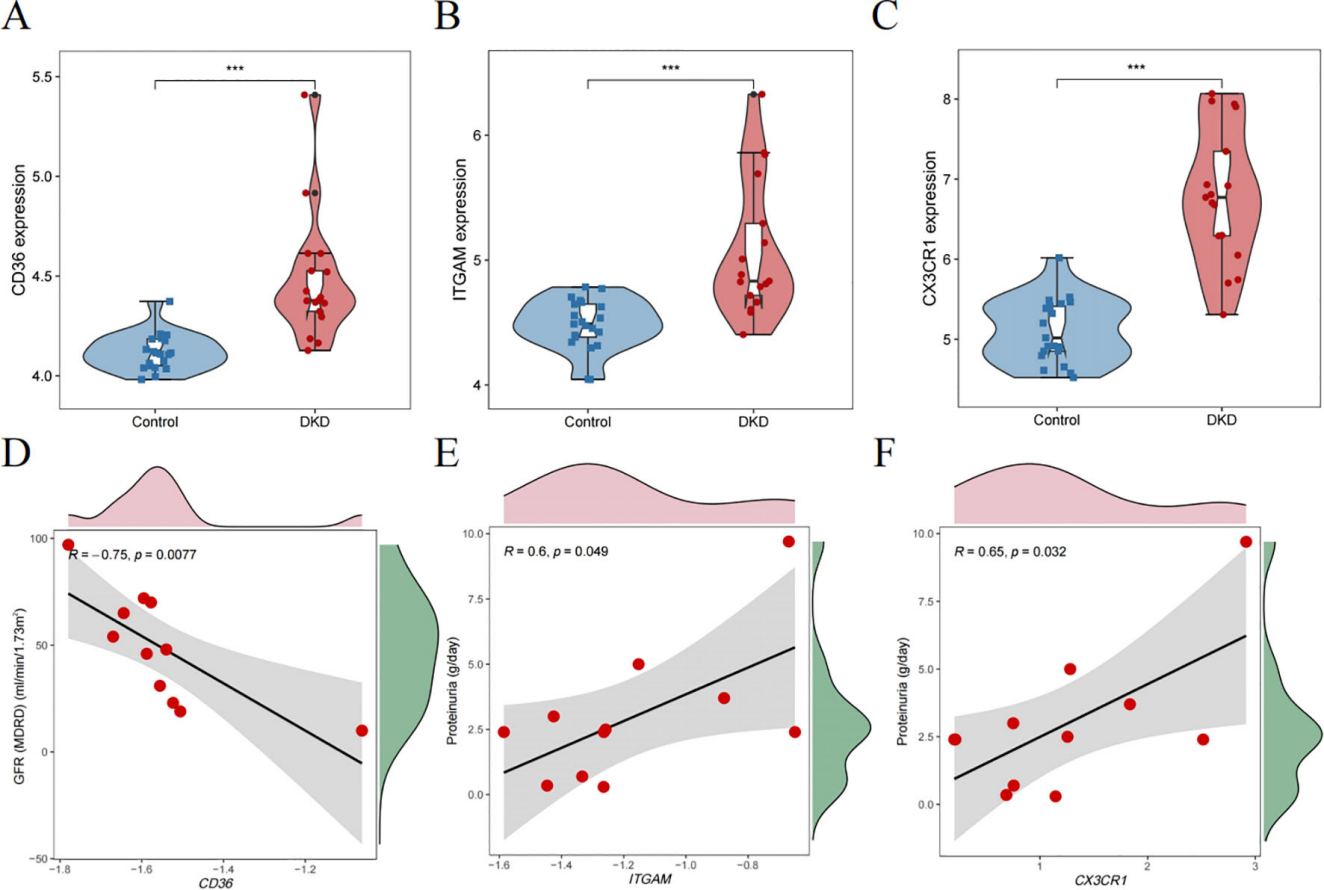
Validating the clinical relevance of hub molecules. **(A–C)** illustrating the distribution characteristics of the data via violin plots. Scatter plots describe the correlation between CD36 **(D)**, ITGAM **(E)**, and CX3CR1 **(F)** expression with clinical indicators. ***P<0.001.

**Figure 9:**
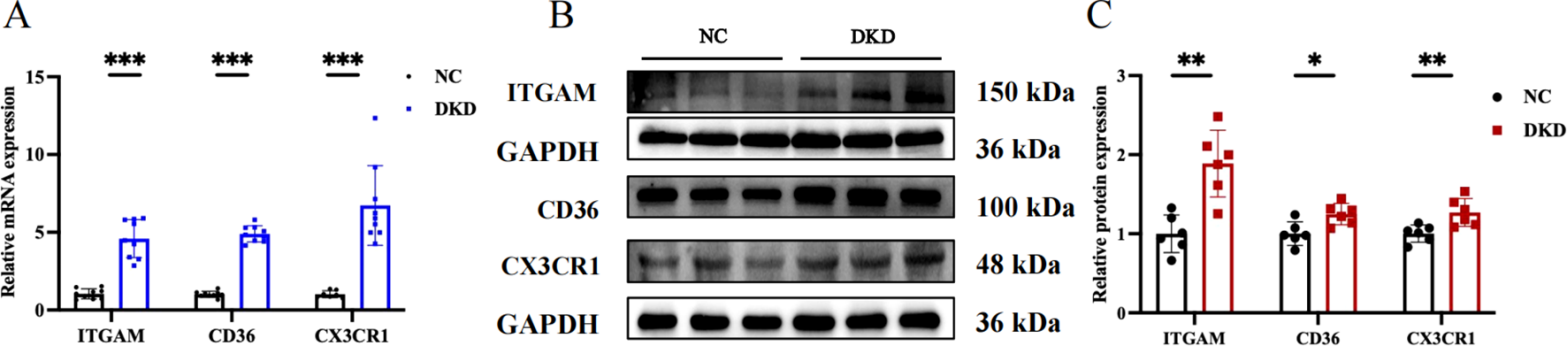
Evaluation of target gene expression in DKD model. **(A)** Quantitative PCR analysis of ITGAM, CD36, and CX3CR1 mRNA expression. **(B)** Western blot showing the protein expression levels of ITGAM, CD36, and CX3CR1. **(C)** Quantitative analysis of protein band grayscale values for ITGAM, CD36, and CX3CR1. *P < 0.05, **P < 0.01, ***P<0.001.

We chose GSE104954 as the external validation dataset, and in line with the training set results, the hub gene showed a similarly increased level in diabetic kidneys, demonstrating the model’s robust assessment performance (**Figures 8A–C**). Following this, we turned to the Nephroseq database to gauge the prognostic importance of key molecules. An increase in CD36 mRNA deposits in the renal tubulointerstitial corresponded to a downward trend in GFR (r = -0.75, p = 0.008). Overexpression of ITGAM (r = 0.60, p = 0.049), and CX3CR1 (r = 0.65, p = 0.032) was positively linked to proteinuria in DN (**Figures 8D–F**). Aligning with database outcomes, our research employing qPCR and Western blot assays has identified a significant upregulation of hub gene expression in DKD kidney tissues (**Figures 9A–C**).

Altered Macrophage Behavior and Efferocytosis Decline in DKD

**Figure 10:**
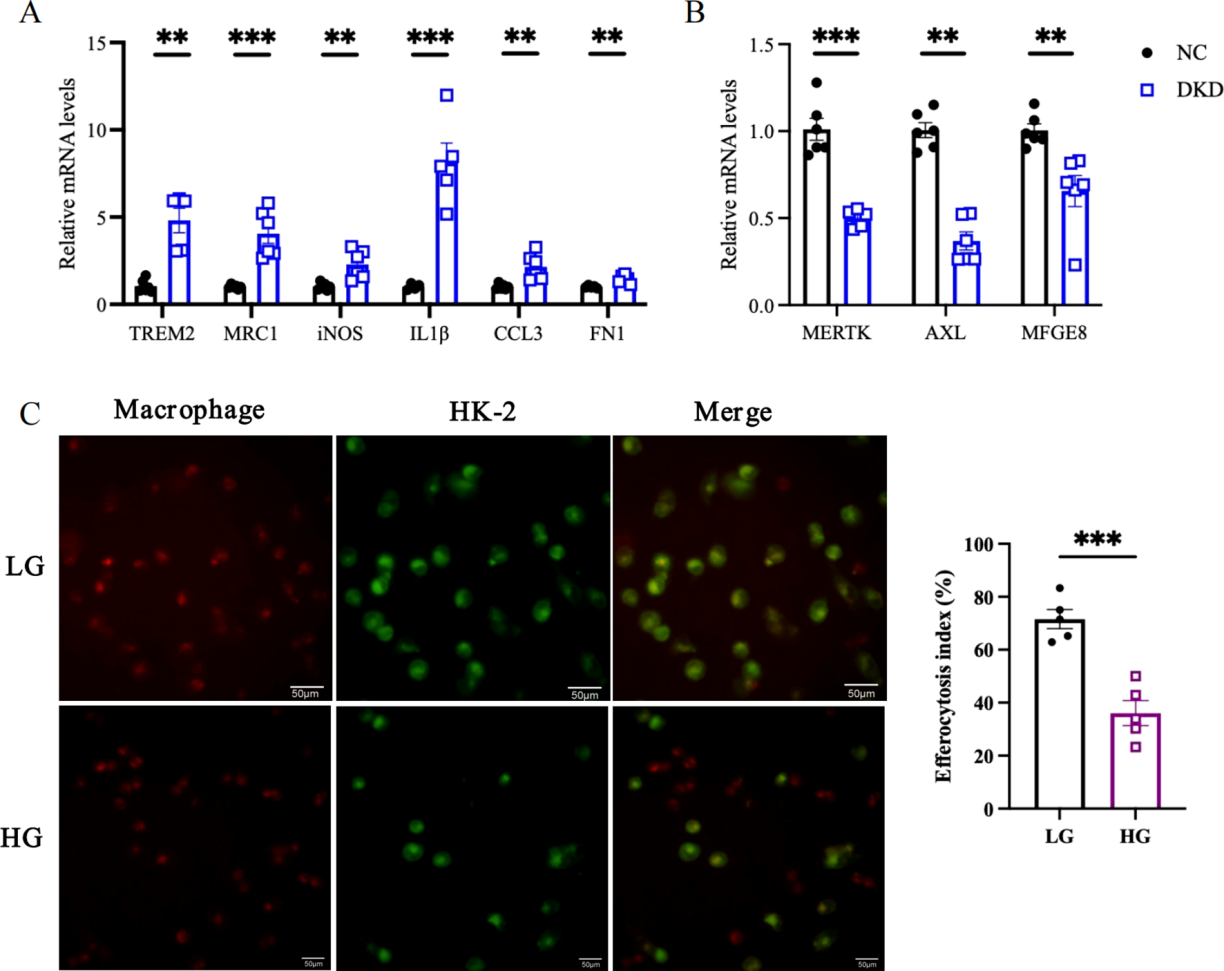
Macrophage dynamics and efferocytosis decline in DKD. **(A)** TRME2, MRC1, iNOS, IL1Β, CCL3, and FN1 mRNA levels detected by qPCR. **(B)** Quantification of MERTK, AXL, and MFGE8 mRNA levels measured through quantitative PCR. **(C)** Under fluorescence microscopy, macrophages (red) are seen to have ingested apoptotic cells (green), with some macrophages remaining unbound. Scale bar represents 50μm. The efferocytosis index, calculated as the proportion of macrophages that have phagocytosed apoptotic cells relative to the total macrophage population. LG, low glucose; HG, high glucose. HK-2, human proximal tubular epithelial cells. **P < 0.01, ***P<0.001.

According to prior analyses, OVE26 mice experienced greater infiltration of Trem2^hi^, Mrc1^hi^, inflammatory, IFN^hi^, and Fn1^hi^ macrophages in the kidney (**Figure 3**). Similarly, in the diabetic mouse model induced by a high-fat diet combined with STZ, we observed upregulated expression of Trem2, Mrc1, iNOS, IL-1Β, CCL3, and Fn1 (**Figure 10A**). Additionally, we detected a decline in the expression of efferocytosis-related molecules MERTK, AXL, and MFGE8 in DKD (**Figure 10B**). The phagocytic capability of macrophages to engulf apoptotic cells was also diminished after high glucose exposure (**Figure 10C**).

## Discussion

Hyperglycemia triggers the innate immune system, setting off an inflammatory cascade (26, 30), and the progressive intensification of inflammatory and immune responses becomes a crucial propelling factor of DKD. As critical modulators of immune reactions, macrophages’ polarization is affected by the local tissue microenvironment. Employing single-cell and bulk RNA-sequencing techniques, this research delves into the distinctive destinies, roles, and dynamic evolutionary traits of renal macrophages (16). The mazy interplay between macrophages and renal cells, along with other immune cells, is orchestrated by a multitude of cytokines, chemokines, and exosomes, creating an inflammatory milieu (30), leading to pathological damages such as glomerulosclerosis, tubular atrophy, and interstitial fibrosis (4, 5).

snRNA-seq affords enhanced resolution to uncover the heterogeneity in tissues, as nuclei maintain relative stability across the cell cycle, resulting in more reliable gene expression patterns. This current research identified 11 distinct cell types in human diabetic kidney sequencing data (14), encompassing various interactions between immune cells and intrinsic renal cells, such as signaling via CCL, CXCL, FN1, and TGFΒ pathways. Prolonged hyperglycemia initiates macrophage polarization, eliciting cytokine release and a chain of reactions. This chronic inflammation, if left unresolved, results in excessive extracellular matrix deposition and kidney fibrosis.

Macrophages exhibit diversity in their properties and functions based on the microenvironment, playing a Janus-faced role in both maintaining host defense and causing tissue damage. However, the traditional classification of macrophages falls short of capturing their full spectrum and dynamic shifts within diabetic kidneys. Consequently, we harvested CD45-enriched renal immune cells at various moments of the disease’s evolution to conduct a thorough analysis of the transcriptional diversity within macrophage subsets (16). Embryonic-origin resident macrophages and bone marrow-derived infiltrating macrophages persistently coexist in the mouse kidneys (31). In the initial phase of DKD, resident macrophages, functioning as the sentinels of immune inflammatory responses, are triggered swiftly. Elevated levels of adhesion molecules in the bloodstream prompt the circulating monocytes into the renal tissue (32). By the age of seven months, there was a substantial decrease in resident macrophages, with a concurrent sharp rise of Mrc1^hi^ and Trem2^hi^ Mac. The buildup and phenotypic shift of macrophages in the kidneys are closely associated with interstitial fibrosis (4). Additionally, we noted the presence of Fn1^hi^ Mac subtype, a novel cell type not seen in three-month-old mice. Furthermore, we applied the MacSpectrum platform to analyze the characteristic distribution of each macrophage subpopulation, calculating the polarization and differentiation index (19). In line with earlier observations (6), the advancement of DKD is marked by a decline in the M1 and an increase in M2 macrophages, with fewer cells in the “pre-activation” state.

Within the tissue immune microenvironment, there is a mutual coordination among DC, macrophages, T cells, B cells, and NK cells. DC are viewed as the initiators of adaptive immune responses and functionally resemble macrophages in preserving renal homeostasis. They can also mobilize effector T cells (33). Activated T cells exhibit cytotoxic effects, and B cells’ functions are not confined to antibody production alone. Both lymphocytes assist in recruiting macrophages and maintaining the equilibrium of immune responses (34).

Macrophages exhibit distinct molecular mechanisms and signaling pathways for their phenotypic specialization, with a distinct divergence between M1 and M2 types (26). M2 macrophages, on one hand, rely on efferocytosis to clear apoptotic cells, and on the other, can undergo a macrophage-to-myofibroblast transition under TGFΒ stimulation (35). Efferocytosis is a widespread physiological process that appears complex, encompassing a range of receptors, cytosolic signaling molecules, swift cytoskeletal reorganization, digestion of cellular contents, and immune regulation (27). Dysfunctions or modifications are observed in various disorders, such as atherosclerosis, diabetes, obesity, cancer, and systemic lupus erythematosus (27).

Furthermore, impaired efferocytosis is considered one of the key contributing factors to diabetic complications (10), resulting in apoptotic cells accumulating in tissues and expanding local inflammation. The receptor for advanced glycation end products (RAGE) interacts with phosphatidylserine on apoptotic cells to prime “eat me” signals (36). In diabetes, elevated AGE levels inhibit cytoskeletal rearrangement through RAGE/Rho kinase signaling (37). Moreover, PARylated high mobility group box 1 exhibits a higher affinity for RAGE, effectively blocking efferocytosis (38).

The DEGs from distinct macrophage subpopulations overlapped with genes participating in efferocytosis, and a PPI network was built to screen for crucial targets. Following this, we performed GSEA on the bulk RNA dataset, with significant pathway enrichments observed in the phagosome, cell adhesion molecules, chemokines, and NOD-like receptor signaling pathway. We then intersected these findings and utilized LASSO regression and ROC curves to ultimately pinpoint the hub genes (CD36, ITGAM, and CX3CR1). They show a correlation between the progression of proteinuria and the deterioration of kidney function, and they may serve as potential key targets driving efferocytosis.

The scavenger receptor, commonly referred to as CD36, is a single-spanning transmembrane glycoprotein found on the surfaces of cells such as monocytes, macrophages, endothelial cells, smooth muscle cells, and platelets in mammals. It features various ligand-binding sites and undergoes post-translational modifications that significantly affect its affinity for ligands, thereby initiating diverse biological responses. CD36 dysfunction is implicated in disturbances of fatty acid metabolism, inflammatory responses, and macrophage polarization. During chronic kidney disease, CD36 expression is elevated, and the phagocytosis of apoptotic cells driven by CD36 could be a crucial route for fibrosis advancement (39). Blocking CD36-induced lipid accumulation and NLRP3 inflammasome can diminish cell apoptosis in DKD, making CD36 a potential therapeutic target for delaying disease progression (40). Another study indicates that CD36 is a substrate for matrix metalloproteinase 9 (MMP9), and its degradation affects the phagocytic activity of macrophages (41).

Macrophage antigen-1 (Mac-1), also known as ITGAM/ITGB2, is a primary member of the integrin Β2 subfamily and is present on the surfaces of phagocytic and natural killer cells. It interacts with numerous ligands to facilitate cell adhesion and inflammatory responses (42). The function of Mac-1 can varies, with different ligands leading to opposing outcomes. The blockade of CD47-SIRPΑ can enhance Mac-1 expression, accelerating the adhesion between macrophages and cancer cells and boosting the subsequent phagocytic process (43). Mac-1 also responds to Listeria infection by initiating LC3-associated phagocytosis (44). Furthermore, ITGAM engages in crosstalk with MyD88 and TRIF pathways, suppressing Toll-like receptors signaling in the innate immune response (45). The ITGAM agonist LA1, a small molecule allosteric activator, augments the adhesion of Mac-1 to ligands and phagocytic activity (46).

CX3CR1, the CX3C chemokine receptor, binds to its exclusive ligand CX3CL1 to facilitate the movement of immune cells, primarily monocytes and macrophages, to inflammatory sites (47). CX3CR1^+^ macrophages are present in diverse tissues, and their pro-inflammatory or anti-inflammatory actions are modulated by the tissue type, microenvironment, and expression level. The CX3CL1-CX3CR1 signaling pathway additionally regulates the adhesion and is linked to obesity, and type 2 diabetes mellitus (48). It participates in the mobilization of macrophages and their M1/M2 polarization in adipose tissue, which is associated with inflammation and insulin resistance (49). The role of CX3CR1 in renal fibrosis is a matter of controversy. Daniel R. Engel et al. proposed that the high expression of CX3CR1 has a suppressive effect on the advancement of renal interstitial fibrosis, possibly by blocking the gathering of macrophages (50). Jie Du et al. have observed that enhanced expression of CX3CR1 accelerates fibrosis in unilateral ureteral obstruction (51). CX3CR1 also serves as a phagocytic receptor, responsible for the elimination of apoptotic cells. Deficiencies or pharmacological inhibition of CX3CR1 can modulate the homeostasis of liver-infiltrating macrophages, boosting MERTK mediated efferocytosis to promote the resolution of liver ischemia-reperfusion (52). CX3CR1+ macrophages employ phagosomes to process abnormal mitochondria, thereby protecting cardiomyocytes (53).

Hub genes may act as a pivotal link in the inflammatory response of DKD, governing immune cells, inflammatory mediators, and apoptosis. The exact molecular dynamics are still a mystery, yet they hold potential as futuristic targets for enhancing the body’s response to low-grade inflammation.

This study comes with its own set of limitations. The dependability of bioinformatics analysis outcomes is heavily reliant on the integrity of the data sets. Technical biases are inevitable due to the varying experimental designs, sample procurement, and sequencing platforms employed by different research teams. Additionally, while cross-species comparative analysis involving humans and mice offers a broader perspective, the biological traits of mice cannot entirely replicate those of humans. Furthermore, bioinformatics analysis and basic experiments are considered complementary. We have verified the hypotheses derived from bioinformatics results *in vivo* experiments, and researchers are required to delve deeper into the mechanisms based on these findings.

## Conclusion

In a nutshell, this study integrated snRNA, scRNA, and bulk RNA-sequencing data to uncover the crosstalk between macrophages and intrinsic cells in diabetic kidneys, as well as the shift in M1/M2 polarization ratio and subtype functions during progression. We also favorably pinpointed three key genes (CD36, ITGAM, and CX3CR1), considering the anti-inflammatory characteristics inherent to efferocytosis. Macrophages are pivotal in the context of chronic immune-inflammatory responses. In-depth research into their regulatory pathways is expected to provide novel and comprehensive perspectives for DKD.

## Data Availability

The datasets presented in this study can be found in online repositories (https://www.ncbi.nlm.nih.gov/geo/). The names of the repository and accession numbers include GSE195799, GSE131882, GSE30122, and GSE104954. Further details or data can be requested from the corresponding author.
