# Supplementary material for: Unveiling macrophage dynamics and efferocytosis-related targets in diabetic kidney disease: insights from single-cell and bulk RNA-sequencing
Source: Front Immunol. 2025 Feb 19;16:1521554. doi: 10.3389/fimmu.2025.1521554 (PMC11879818; doi:10.3389/fimmu.2025.1521554)
Supplement: Supplementary file 1 [file DataSheet1.docx]

**Supplementary materials**

**Supplementary Figures**


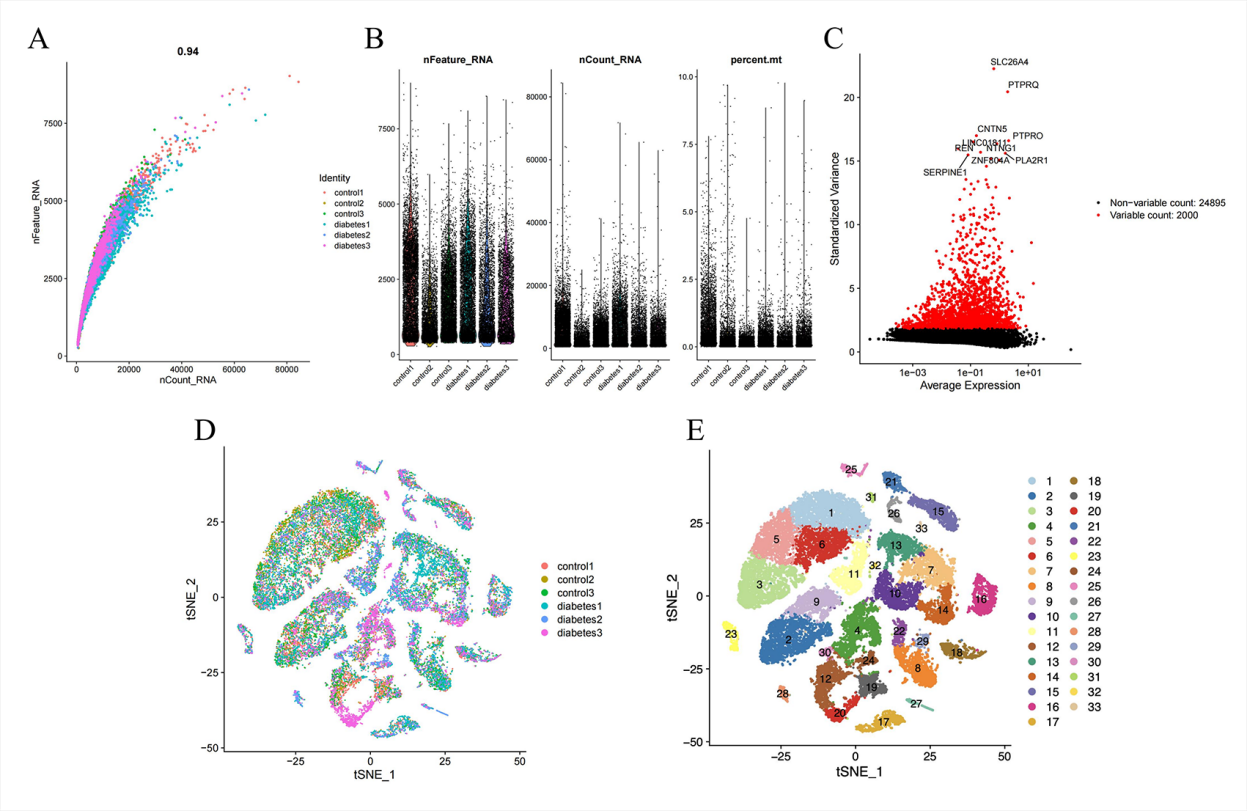


**Figure S1. Data preprocessing.** (A) The interplay between RNA counting and the diversity of genes. (B) A representation of the filtered cell quality. (C) An exhibition of the highly variable genes (HVG). (D) t-SNE diagrams of each sample and 33 clusters (E). t-SNE, t-distributed Stochastic Neighbor Embedding.


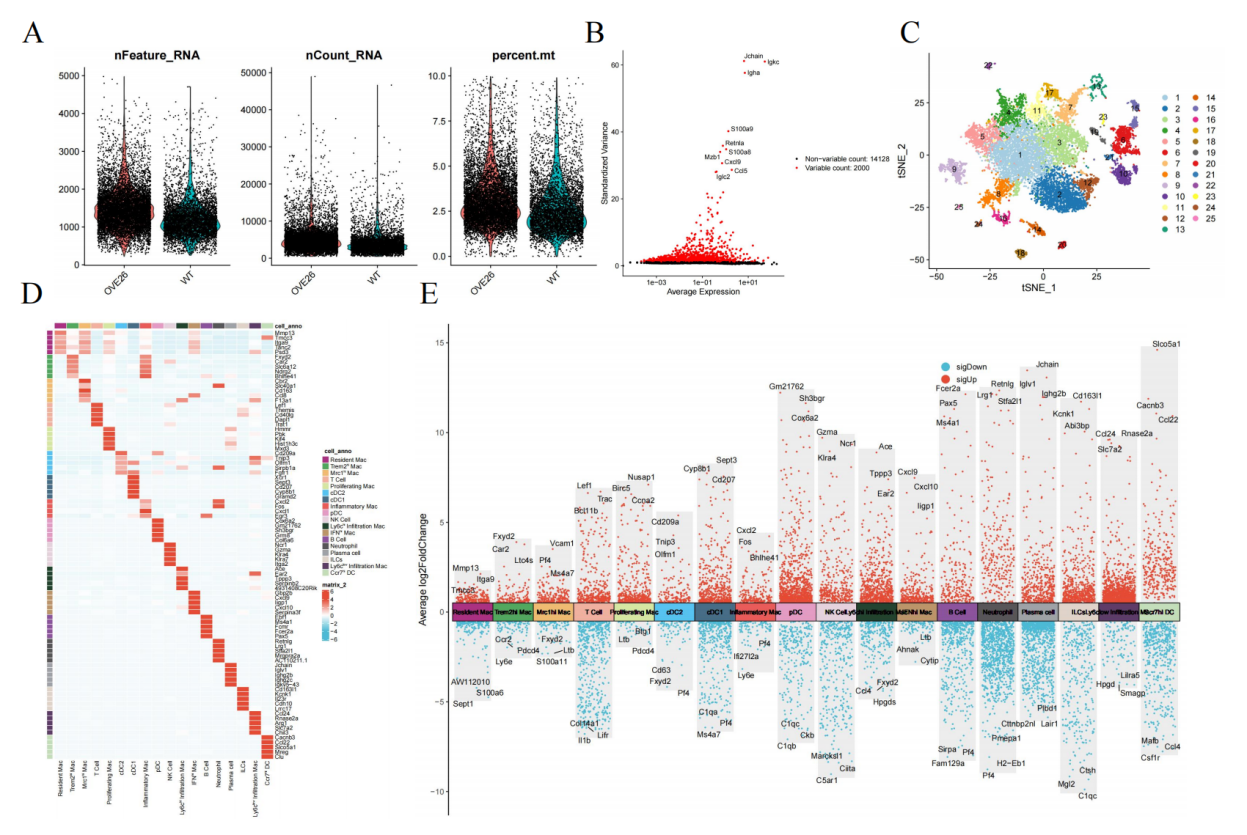


**Figure S2. Preconditioning data matrix of 3-month-old mice.** (A) Violin plots depicting the quality of cells post-QC, including the gene features, counts, and percentage of mitochondrial genes. (B) Label the top 10 most variable genes. (C) t-SNE visualization of 25 clusters. (D) Displays a hierarchical clustering heatmap of the top 5 genes in each subcluster. (E) Significantly regulated genes are displayed through the volcano plot. QC, quality control t-SNE, t-distributed Stochastic Neighbor Embedding.


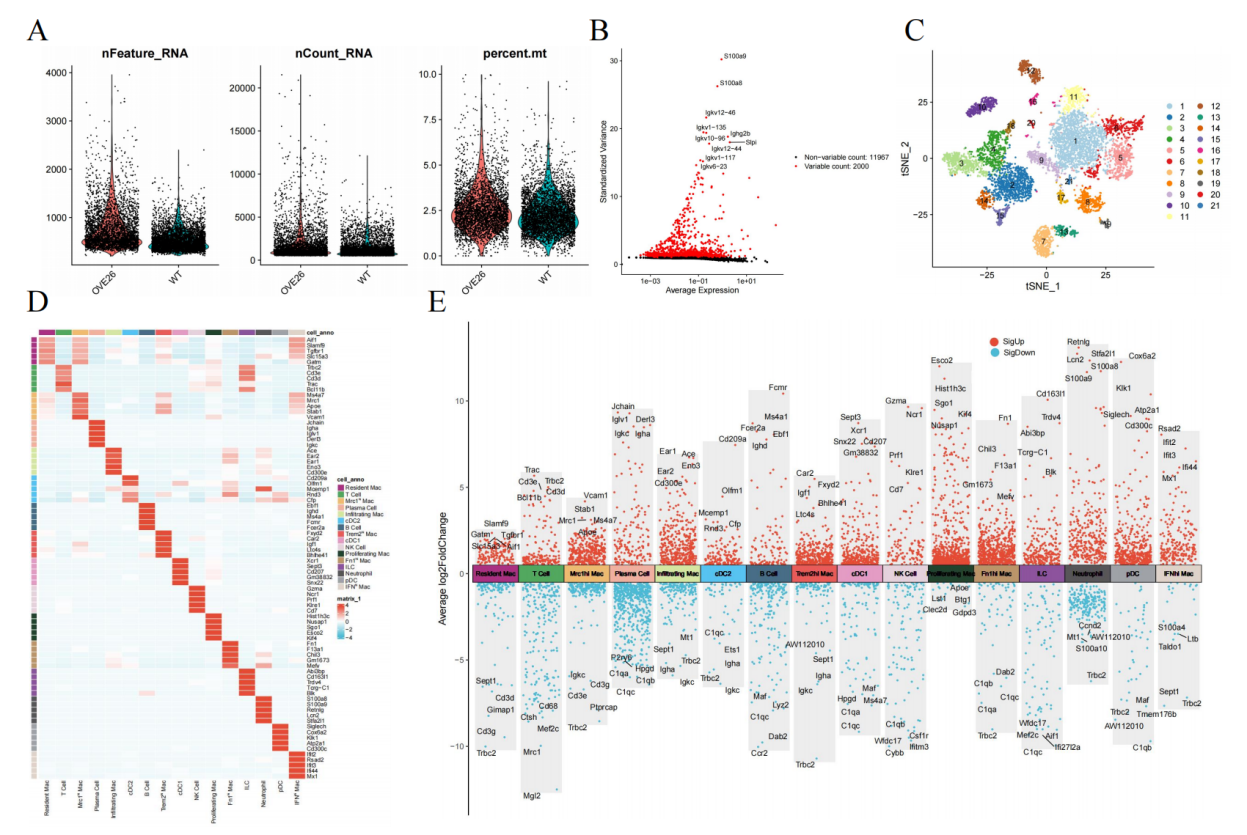


**Figure S3. Preconditioning data matrix of 3-month-old mice.** (A) Violin plots depicting the quality of cells post-QC, including the gene features, counts, and percentage of mitochondrial genes. (B) Label the top 10 most variable genes. (C) t-SNE visualization of 21 clusters. (D) Displays a hierarchical clustering heatmap of the top 5 genes in each subcluster. (E) Significantly regulated genes are displayed through the volcano plot. QC, quality control; t-SNE, t-distributed Stochastic Neighbor Embedding.

**Supplementary Tables**

| Target Gene | Forward (5’ to 3’) | Reverse (3’ to 5’) |
| --- | --- | --- |
| GAPDH | TGTGTCCGTCGTGGATCTGA | AGTGTAGCCCAAGATGCCCT |
| TREM2 | CTGGAACCGTCACCATCACTC | CGAAACTCGATGACTCCTCGG |
| MRC1 | AATGCTGACCTCCTGAGTGT | CAGTTCAGATACCGGAATGG |
| iNOS | GCAGAGATTGGAGGCCTTGTG | GGGTTGTTGCTGAACTTCCAGTC |
| IL-1β | GCCCATCCTCTGTGACTCA | AGGCCACAGGTATTTTGTCG |
| CCL3 | TTCTCTGTACCATGACACTCTGC | CGTGGAATCTTCCGGCTGTAG |
| FN1 | AGTCAGCTGCCAAGAGACAG | ACGTCCTGCCATTGTAGGTG |
| MERTK | CTCCTGAGCCCGTCAATATCT | AGACCAGGTACGGTTAGGACA |
| AXL | ATGGCCGACATTGCCAGTG | CGGTAGTAATCCCCGTTGTAGA |
| MFGE8 | CGGGCCAAGACAATGACATC | TCTCTCAGTCTCATTGCACACAAG |
| CD36 | GCCAGTCGGAGACATGCTTA | TGCCACGTCATCTGGGTTTT |
| ITGAM | GGAACCAGTGTGGTTGTTGC | GAGGTACTTGCAGGGGGATG |
| CX3CR1 | TCTTCACGTTCGGTCTGGTG | GTTGCACTGTCCGGTTGTTC |

**Table S1. Mouse-specific primer pairs used for quantitative polymerase‑chain reaction.**

|  | PCT | DCT | LOH | CT | ICA | ENDO | PEC | ICB | PODO | MES | LEUK | Total |
| --- | --- | --- | --- | --- | --- | --- | --- | --- | --- | --- | --- | --- |
| control1 | 2103 | 1090 | 1361 | 645 | 366 | 486 | 267 | 216 | 121 | 95 | 21 | 6771 |
| control2 | 1958 | 519 | 458 | 562 | 180 | 114 | 74 | 43 | 37 | 28 | 17 | 3990 |
| control3 | 2143 | 883 | 720 | 1271 | 678 | 378 | 154 | 103 | 216 | 78 | 25 | 6649 |
| diabetes1 | 1685 | 631 | 245 | 1225 | 429 | 350 | 230 | 265 | 130 | 78 | 117 | 5385 |
| diabetes2 | 854 | 277 | 616 | 986 | 203 | 237 | 126 | 119 | 82 | 48 | 248 | 3796 |
| diabetes3 | 602 | 380 | 1505 | 564 | 129 | 233 | 84 | 13 | 115 | 33 | 23 | 3681 |

**Table S2. The counts of specific cell types are detailed for each sample.** PCT, proximal convoluted tubule; PEC, parietal epithelial cells; LOH, loop of Henle; DCT, distal convoluted tubule; CT, connecting tubule; ICA, type A intercalated cells; ICB, type B intercalated cells; PODO, podocytes; ENDO, endothelial cells; MES, mesangial cells; LEUK, leukocytes.

| S1pr1 Find me | Timd2 Eat me | Trem2 Eat me | Cdkn2b Engulfment | 1700021K19Rik Engulfment |
| --- | --- | --- | --- | --- |
| P2ry2 Find me | Havcr2 Eat me | Tyrobp Eat me | Mapk7 Engulfment | Becn1 Engulfment |
| G2a Find me | Timd4 Eat me | Slc2a1 Eat me | Hmgb1 Engulfment | Pik3c3 Engulfment |
| Fasl Find me | Cd14 Eat me | Ager Eat me | Il10 Engulfment | Atg5 Engulfment |
| Cx3cl1 Find me | Cd36 Eat me | Tyro3 Eat me | Irf5 Engulfment | Atg7 Engulfment |
| Cx3cr1 Find me | Cd93 Eat me | Axl Eat me | Irf8 Engulfment | Dnm1l Engulfment |
| Panx1 Find me | Cd300lb Eat me | Mertk Eat me | Mapk14 Engulfment | Dnase2a Engulfment |
| Aimp1 Find me | Cd300lf Eat me | Msr1 Eat me | Mapk1 Engulfment | Ctsg Engulfment |
| S1pr2 Find me | Lrp1 Eat me | Marco Eat me | Cdkn1a Engulfment | Nr1h2 Engulfment |
| S1pr3 Find me | Itgb2 Eat me | Itgam Eat me | Sirt1 Engulfment | Nr1h3 Engulfment |
| S1pr4 Find me | Thbs1 Eat me | Clec9a Eat me | Tlr3 Engulfment | Ppard Engulfment |
| S1pr5 Find me | Tgm2 Eat me | Itgb3 Eat me | Tlr9 Engulfment | Pparg Engulfment |
| Calr Eat me | Pros1 Eat me | Apoa1 Eat me | Treml2 Engulfment | Tgfb1 Engulfment |
| Ptx3 Eat me | Stab1 Eat me | Scarb1 Eat me | Traf6 Engulfment | Crk Engulfment |
| Mfge8 Eat me | Stab2 Eat me | Gas6 Eat me | Trpc3 Engulfment | Atg16l1 Engulfment |
| Anxa1 Eat me | Icam5 Eat me | Scarf1 Eat me | Ucp2 Engulfment |  |
| Anxa2 Eat me | Bai1 Eat me | Lgals3 Eat me | Rac1 Engulfment |  |
| Anxa5 Eat me | Megf10 Eat me | Abca1 Engulfment | Elmo1 Engulfment |  |
| Havcr1 Eat me | Gulp1 Eat me | Abca7 Engulfment | Dock1 Engulfment |  |

**Table S3. Efferocytosis-related genes from literature.** Macrophage efferocytosis encompasses three stages, with 12 molecules identified in the "find me" phase, 43 molecules involved in the "eat me" signal, and 36 molecules mediating the "engulfment" process.
